# 暴露组学在识别环境污染物及其健康危害中的应用进展

**DOI:** 10.3724/SP.J.1123.2023.12011

**Published:** 2024-02-08

**Authors:** Mengxue ZHI, Jianshe WANG

**Affiliations:** 烟台大学药学院, 山东 烟台 264005; School of Pharmacy, Yantai University, Yantai 264005, China

**Keywords:** 暴露组学, 污染物, 生物标志物, 健康, 综述, exposomics, pollutants, biomarker, health, review

## Abstract

目前,全球范围内的环境污染成为困扰各国的突出问题,环境污染导致的健康危害已引起世界各国的广泛关注,但揭示繁杂的污染物暴露与相关疾病的关系仍是一个科学难题。暴露组学概念的提出为推动环境因素与人类健康之间关系的研究提供了新思路。“自上而下”和“自下而上”的研究策略、执行研究策略所使用的检测手段以及“组学”的研究共同促进了暴露组学的发展。暴露组学关注个体一生中所有暴露的测量,旨在通过传统生物监测和现代组学方法对体内外暴露水平进行动态监测,考察与所有环境暴露相关的标志物以及与疾病相关标志物之间的相关性,从而得到全面可靠的环境与疾病风险的关系。与传统环境健康研究相比,暴露组学研究更能真实体现现实环境中污染物、自然因素及生活方式等暴露因素的多样性,以及污染物的体内过程和所触发的机体反应的复杂性。高分辨质谱技术等强大的化学分析手段被广泛应用于暴露组学的相关领域研究中,本文简单举例介绍了色谱-质谱联用技术在环境污染物检测和分析中的应用,其中蛋白质组学和代谢组学作为生物标志物鉴定和污染物效应分析的两个重要手段,在探究环境与疾病的联系中具有重要意义,并得到广泛的应用。进入机体的污染物,可与机体(尤其是与生物大分子中的蛋白质)发生复杂的相互作用,导致病理改变和毒性效应;在混合暴露的情况下,污染物间也会存在相互作用,影响各自的环境行为或进入人体的量,进而影响其健康效应。

## Abstract (en)

Environmental pollution has become a prominent global problem, and the potential health hazards of pollutants have caused widespread concern. However, revealing the relationship between complex-pollutant exposure and disease development remains an immense challenge. The core of environmental-health research and risk assessment is the identification of contaminants and their effects. Exposomics provides a new approach in the study of the relationship between environmental factors and human health. Both “top-down” and “bottom-up” strategies are employed in exposomics research. The development of new technologies for chemical detection and “multi-omics” has greatly facilitated the implementation of these strategies. Exposomics focuses on the measurement of an individual’s lifelong exposure and aims to identify the health effects of such exposure. It involves the dynamic monitoring of external and internal exposure levels at different stages of life through traditional biomonitoring and exposomic methods. It also includes the identification of biomarkers, which indicate specific environmental exposures and the adverse effects of these exposures on health. Compared with traditional environmental-health studies, exposomics can more accurately reflect the diversity of exposure factors such as pollutants, natural factors, and lifestyles in the real environment, as well as the complexity of their in vivo processes and the responses they trigger in an organism. Powerful chemical analytical tools such as high-resolution mass spectrometry (HRMS) are widely used in studies related to the field of exposomics. Liquid chromatography-mass spectrometry (LC-MS) has been applied in the detection and analysis of environmental pollutants. Proteomics and metabolomics, as two important tools for biomarker identification and effects analysis, are widely used to explore the relationship between environmental factors and diseases. Pollutants can lead to pathological changes and even toxic effects by interacting with proteins. In the case of mixed exposure, some contaminants may present joint toxicity. The interaction between contaminants may change their environmental behavior or the amount of each contaminant that enters the human body, which, in turn, affects their health effects.

健康或疾病是由环境因素和遗传因素共同作用的结果。随着人类基因组计划的完成和对复杂疾病病因学研究进展的加快,科学家发现遗传因素仅占疾病病因的10%左右,大多数疾病是由非遗传因素导致的,环境暴露在人体健康与疾病中起到重要的作用^[1]^。2016年,世卫组织报告称,全球有1260万例死亡可归因于环境,占总死亡人数的23%。如果将死亡和残疾都计算在内,环境造成的疾病负担占全球疾病负担的22%^[2]^。因此,要了解疾病发生的原因并最终预防疾病,就需要重视环境因素。结合全基因组关联研究(genome-wide association studies, GWAS)与全暴露(或叫全环境)组关联研究(exposome/environment-wide association studies, EWAS),找出疾病与遗传、环境的联系,通过消除/避免病因来提高预测个体发病风险的能力^[3]^。

## 1 暴露组学概述、研究策略与方法

### 1.1 暴露组学概述

全球经济和人口扩张带来了土壤问题、大气问题、全球变暖和水资源危机等一系列生态和环境问题,这些问题不仅影响了我们的生活质量,还严重影响了人民的生命健康。为引起人们对复杂疾病中环境因素的关注,美国癌症流行病学家Christopher Wild于2005年首次提出“暴露组”的概念,他将暴露组定义为从受精卵开始涵盖整个生命周期的所有环境暴露,包括生活方式^[4]^。这一概念用来表示健康和疾病的环境驱动因素,即非遗传驱动因素。此后,“暴露”的定义进一步细化为对整个生命周期中环境影响和相关生物反应的累积测量^[5⇓-7]^,其中生物效应是通过改变体内关键分子、细胞和生理过程的化学物质介导的,所以暴露不仅限于从空气、水或食物进入体内的化学物质(有毒物质),还包括炎症、氧化应激、脂质过氧化、感染、肠道菌群和其他过程产生的化学物质。因此,Wild于2012年将“暴露”分为3类:内部暴露、一般外部暴露和特定外部暴露。内部暴露被定义为个体对环境刺激的反应或维持体内平衡所需的生理和生物反应,包括炎症、代谢和应激途径等。宏观层面的暴露,如涉及物理或社会环境的暴露(如空气和水、建筑环境、气候变化和噪声)构成一般外部暴露,而个人层面的暴露(如沐浴/化妆品使用、饮食、生活方式、体育活动和睡眠)代表特定外部暴露^[8,9]^。随之出现了暴露组学的概念:“研究暴露组以及暴露组对人类疾病过程影响的学问”^[10]^。暴露组的研究补充了基因组学在人类复杂疾病病因研究中的不足。

### 1.2 暴露组学的研究策略和方法

为研究环境对疾病和健康的影响,针对复杂的环境暴露和更复杂的人对环境的反应,提出了“自下而上”和“自上而下”两种研究策略。“自下而上”的研究策略是通过对空气、水和食物等介质中的化合物进行测定,分析他们与疾病和健康结果的相关性,寻找确定影响疾病的外源性暴露因子。“自上而下”的策略通过分析测定患者和健康人群血液或者尿液等生物样本中所有外来化合物的种类和水平,应用高通量的组学技术对人体内的标志物进行分析,识别所有可能的暴露因素,分析其与疾病的关联,确定导致健康损害的有害因子(见图1)。“自下而上”与“自上而下”两种策略各具优势和不足,前者能在众多的有害因素中分析其环境介质的来源,可以进行大规模的人群研究,但是难以分析进入体内有害物质的量,不能得出与疾病的明确关系;后者由于可以测定进入体内的有害因子含量及其效应的标志物,为确定与疾病的关系提供了有力证据,但是由于生物材料的采集和分析的限制,难以进行大规模的人群研究,并且也无法确定有害因素的来源^[1,11]^。两种方法相结合可发挥暴露组学方法的最大应用潜力^[12]^。

**图1:**
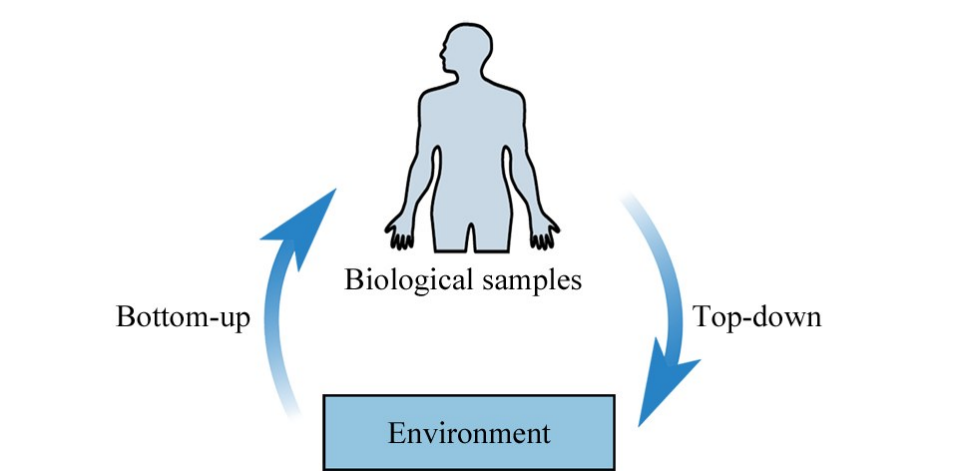
暴露组学研究的基本策略

外部因素通过改变机体的生物学特征对健康产生影响。通过分析不同生物样本的化合物水平,并结合暴露时体内生理学变化和体外暴露因素信息来理清暴露因素和疾病的联系,可在个人和社会层面采取干预措施,降低健康风险。暴露组学的研究依赖于内部和外部暴露评估方法的应用。外部暴露评估依赖于对环境压力源暴露量的测量,内部暴露评估依赖于基因组学、代谢组学、脂质组学、转录组学和蛋白质组学等组学研究^[13⇓⇓⇓-17]^。近些年来组学方法取得了非凡的进步,使毒理学家能够将毒代动力学和毒效动力学与有毒化学物质作用方式(单一或联合)的机制结合起来,为系统评估环境污染物的健康风险提供理论依据^[18,19]^。基因组学被认为是组学分析的基础,即使微小的基因突变都可能导致个体的毒物反应差异,影响个体对疾病的易感性^[15]^。表观遗传学研究基因甲基化和染色质构象改变等方式引起的基因表达调控变化和最终导致的表型改变。转录组学关注RNA水平上的基因表达,使我们能够在转录水平上了解基因组的表达,从而提供基因结构、基因表达调控、基因产物功能和基因组动力学等方面的信息^[15]^。与其他组学相比,蛋白质组学更接近生物表型^[20]^。机体在外界刺激下,表现出不同的蛋白质表达水平,这些信息将有助于了解蛋白质对表型的影响以及疾病发生的途径或机制^[15]^。代谢组学通过获取的内源或外源小分子物质变化谱可以反映有害暴露下的细胞活动改变^[21]^,敏感、特异性内源代谢物变化可以作为生物标志物来指示暴露因子的性质和潜在危害^[14,20]^。上述任何一种组学分析在全面解释特定生物现象方面都可能存在局限性,“多组学”方法通过整合多种组学技术,从不同生物层次识别与有害结局相关的生物学通路,以全局的角度研究一系列生物效应,找出组内关联,从而在分子水平上加强对环境影响和健康结果的精确理解,确认环境暴露与发病机制之间的因果关系和关联^[15]^。

## 2 暴露组学在识别环境污染物及健康危害中的应用

### 2.1 暴露组学在环境污染物识别中的应用

近些年新技术的发展显著促进了对化合物的检测和识别能力,通过分析获得的丰富数据促进了未知化合物的发现。其中高分辨质谱法(high-resolution mass spectrometry, HRMS)已成为表征暴露水平和发现暴露相关生物途径变化或寻找疾病标志物的重要手段^[22]^,是研究持久性有机污染物(POPs)、内分泌干扰化学物质、农药、重金属和空气污染物等各种环境风险因素及其效应的强大工具^[23,24]^。例如,一项研究^[25]^采用超高效液相色谱-串联质谱法(UPLC-MS/MS)和液相色谱-四极杆飞行时间质谱(LC-QTOF-MS)对水源水和自来水中的全氟/多氟烷基化合物(PFASs)进行靶向、非靶向和可疑筛选,在自来水中靶向检出12类共50种PFASs;非靶向检测出15种PFASs,总含量高达17.6 ng/L,有3种高置信度PFASs首次在饮用水中检出;对应的水源水和自来水中靶向PFASs的浓度变化不明显,说明常规饮用水处理方式对这些PFASs的去除效率较低。另一项研究^[26]^采用超高效液相色谱-四极杆线性离子阱质谱和UPLC-QTOF-MS分析方法,对南海海洋哺乳动物肝脏样本中的PFASs进行靶向、非靶向和可疑筛选,发现在21种目标PFASs中,全氟辛烷磺酸(PFOS)和6∶2氯化聚氟烷基醚磺酸(6∶2 Cl-PFESA)占主导地位;非靶向分析和可疑筛选共获得9类44种PFASs,其中有15种PFASs为首次在海洋哺乳动物体内检出。此外,大气压光电离(APPI)技术对芳香族化合物具有很高的灵敏度^[27]^;傅里叶变换离子回旋共振质谱法(FT-ICR MS)具有超高分辨率和质量精度,广泛用于探索复杂材料的分子组成^[28]^;全二维气相色谱(GC×GC)具有分辨率高、峰容量大、灵敏度高等优点,其与飞行时间质谱(TOF MS)联用,在提高分辨率和灵敏度的同时,能减少基质干扰的影响^[29]^。Xu等^[28]^采用大气压光电离傅里叶变换离子回旋共振质谱法(APPI FT-ICR MS)和全二维气相色谱-飞行时间质谱联用(GC×GC-TOF MS)方法,对北京地区大气颗粒物(PM)中的多环芳香类化合物(PACs)进行分析,采用APPI FT-ICR MS对PACs进行初步定性分析,GC×GC-TOF MS进一步鉴定PACs的结构,共识别出386种PACs,其中283种多环芳烃(PAHs)及其烷基衍生物为优势化合物,除包括常规监测的16种PAHs之外,多种PAHs烷基化衍生物以较高浓度存在于PM中^[28]^。李想等^[30]^基于固相萃取-气相色谱-串联质谱技术(SPE-GC-MS/MS)对武汉市普通人群血清中有机氯农药与多氯联苯进行检测分析,张续等^[31]^采用固相萃取-超高效液相色谱-串联质谱法(SPE-UPLC-MS/MS)对尿液中多种农药及农药代谢物进行批量分析测定,两种方法均具有操作简单、灵敏度高的特点,适合大量样本的生物监测。表1简单列出了近年来暴露组学在环境污染物识别中的应用。

**表1:** 暴露组学在环境污染物识别中的应用

Sample matrices	Detection methods	Analytes	Ref.
Dianshan lake water	UPLC-HRMS	pesticides, drugs, plastic additives, surfactants	[32]
Complex feed	UHPLC-MS/MS	>1200 biotoxins, pesticides, veterinary drugs	[33]
Cereals	UHPLC-MS/MS, GC-MS/MS, AAS	mycotoxins, pesticide residues, heavy metals	[34]
Drinking water	UPLC-MS/MS, LC-QTOF-MS	PFASs	[25]
Fruits and vegetables	GC-ECD	pesticides	[35]
Particulate matter (PM)	APPI FT-ICR MS, GC×GC-TOF MS	PACs	[28]

AAS: atomic absorption spectrometer; GC-ECD: gas chromatography with electron capture detection; APPI FT-ICR MS: atmospheric pressure photo-ionization Fourier transform ion cyclotron resonance mass spectrometry. PFASs: per- and polyfluoroalkyl substances; PACs: polycyclic aromatic compounds.

### 2.2 暴露组学与生物标志物的识别

环境暴露对人体的影响贯穿一生,他们影响基因的表达、训练和免疫系统的塑造,触发许多生理反应,决定机体健康和疾病状态。只有阐明环境暴露与各种细胞成分之间复杂的相互作用,才能表征暴露物及其对健康的影响,确定因果关系^[36]^。为了更好地了解复杂化学混合物的暴露情况并保护公众健康,确定暴露对生物的影响尤为重要,这种影响包括生物效应(即暴露的不利影响)和生物反应(即机体试图修复暴露所致损伤做出的反应)^[36⇓-38]^。生物标志物是生物体内与暴露或疾病相关联的指示物,是机体由于接触各种环境因子所引起的器官、细胞、亚细胞水平变化的生化、生理、免疫和遗传学等指标,是一个在暴露组学中广泛应用的概念,从功能上一般分为暴露(接触)生物标志物、效应生物标志物和易感性生物标志物^[39]^。暴露生物标志物能比较准确地反应机体的负荷或吸收的总剂量,包括可直接测量的化学物质(如血液中的外源污染物)或是通过生理机制以各种方式修饰过并仍能识别的化合物(如代谢产物或加合物),用于指示污染物类型和暴露量^[40]^。效应标志物指机体中可测出的生化、生理、行为或其他改变的指标,表明环境污染的致病机制,并提高对疾病的预测和预警的精确度^[1]^。多种重要通路可以通过多层次组学标志物(DNA、RNA、蛋白质和代谢产物)来表征,包括氧化应激、炎症、免疫反应改变和激素调节等^[39]^。暴露生物标志物与效应标志物可用于对剂量-反应(效应)关系进行评价,了解内/外暴露与疾病之间的关联程度^[41]^。易感性生物标志物是机体接触某种特定环境因子时,指示个体反应能力的先天性/获得性缺陷的指标,可用于筛选环境暴露的敏感人群^[1]^。总之,生物标志物在揭示环境暴露、人类生物学和疾病之间的关系方面发挥着重要作用(见图2)。

**图2:**
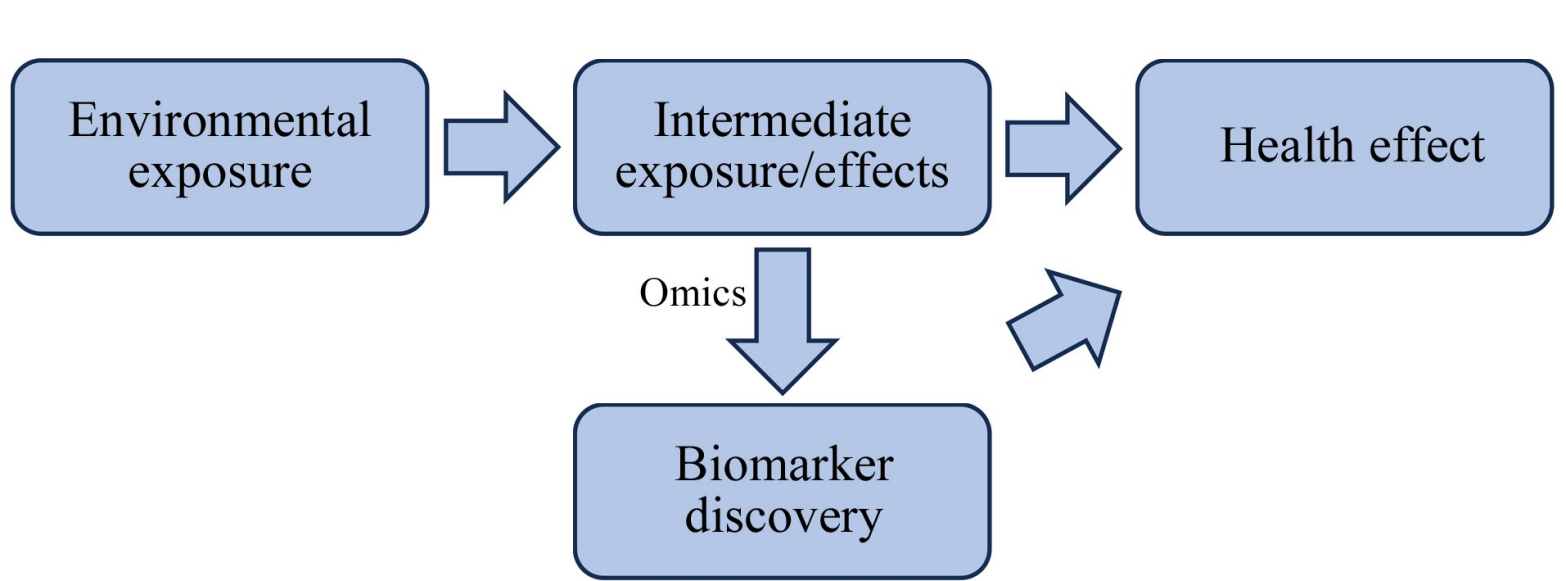
环境暴露与健康效应的关系

暴露组学旨在同时考察所有与环境暴露相关的标志物以及与疾病相关标志物之间的相关性,从而得到全面可靠的环境与疾病风险的关系。研究的难点在于如何一次性考察复杂生物样品中所有环境暴露相关标志物以及疾病相关标志物^[42]^。蛋白质组学和代谢组学是标志物鉴定和污染物效应分析的两个主要手段。蛋白质组学技术通过对蛋白质高效快捷的定量分析,可从蛋白质水平上研究外源性化合物对机体的毒性作用机制,并从中筛选出具有较高特异性和灵敏度的蛋白质标志物^[43]^。同位素标记相对和绝对定量技术(iTRAQ)具有测定蛋白质范围广泛、检出限低、分析结果可靠、精度高等优点^[44]^。基于iTRAQ LC-MS/MS方法,Liu等^[45]^对暴露于全氟辛酸(PFOA)的SD大鼠肝脏进行了差异蛋白质组学分析,确定了3327个非冗余蛋白质,其中192个蛋白质在PFOA处理下呈现显著的改变。与传统的LC-MS/MS相比,纳米液相色谱-串联质谱分析法(Nano LC-MS/MS)在灵敏度上具有更大优势^[46]^, Li等^[47]^采用亲水作用液相色谱(HILIC)富集,Nano LC-MS/MS和无标记定量(LFQ)相结合的研究方法,对PFOS暴露的小鼠肝脏进行蛋白质组学分析,识别出2439种蛋白质和799种糖蛋白,前者有241个发生显著改变(112个上调,129个下调);后者有134个为差异糖蛋白(60个显著上调,74个显著下调)。代谢组学是确定污染物暴露的靶器官、生物标志物和高效解析其毒理作用及机制的重要技术手段^[48]^。靶向代谢组学专注于对一类特定代谢物的定量,特别是针对某一条通路的关键代谢物,可用于对化合物进行毒性评价;非靶向代谢组学专注于对代谢物的全面检测,更适合全面探究暴露因子作用下代谢谱的改变和作用机理^[49,50]^。超高效液相色谱-四极杆-飞行时间质谱法结合了超高效液相色谱和高分辨质谱的优点,是一种快速、灵敏度高、分离能力强、数据采集准确的分析技术,被广泛地应用于复杂化学成分的结构鉴定^[51]^。Chu等^[52]^采用UPLC-QTOF-MS分析方法测定血浆代谢组学,评估长期暴露于PM与血浆代谢变化之间的关系。在PM10和PM2.5暴露下,分别检测到124和162种代谢物。PM2.5暴露下鉴定出的25种差异代谢物大多数是磷脂分解产物,提示长期暴露于PM2.5可能影响磷脂分解代谢;对比PM2.5易感人群和普通人群,在易感人群中确认了6种差异代谢物,其中有2种在空气净化器干预前后发生显著变化,这2种代谢物可作为PM2.5暴露的潜在生物标志物。

### 2.3 污染物与机体的相互作用

污染物对健康的挑战不仅在于需明确日常生活中大量接触化学物质的类型、剂量、频率和持续时间,还在于他们与机体复杂的相互作用。传统毒理学研究主要集中在污染物对生物体的剂量-反应关系和毒性终点指标上。然而,生命体由碳水化合物、脂质、核酸和蛋白质等生物大分子构成^[53]^,这些生物大分子之间复杂的相互作用是生命体生长、发育和存在的基础。污染物对生命健康的危害经常是通过与体内生物大分子的相互作用来实现的,因此进一步在分子水平上研究污染物与生物大分子的相互作用必不可少^[54,55]^。本文以广受关注的POPs为例简要阐述污染物与生物大分子的相互作用。POPs能广泛、持久地分布于各种环境介质和生物体内,很多还具有生物积累和生物放大效应^[56]^,能造成神经行为障碍^[57]^、致癌^[58]^和激素失衡^[59]^等不良后果,严重威胁人类健康。有些POPs与体内的配体或代谢物等物质的结构相似,与蛋白质、核酸、脂质和碳水化合物等生物大分子具有亲和力^[60]^,进入机体后会首先作用于生物大分子,改变生物大分子的结构,从而导致一系列的病变。如PFASs与DNA相互作用会诱导DNA变形^[61]^, PFOA与脂质双分子层的磷脂相互作用能破坏细胞膜结构,诱导细胞死亡^[62,63]^。与核酸和碳水化合物等其他生物大分子相比,蛋白质与污染物的相互作用更普遍,POPs通过氢键、范德华力以及疏水相互作用力与蛋白质靶点(包括酶、血清蛋白、转录因子、转运蛋白和G蛋白偶联受体(GPCRs)等)相互作用。从污染物的代谢动力学角度来看,研究污染物与蛋白质的相互作用有助于在分子水平上了解POPs的吸收、分布和代谢^[64]^。在效应学角度来看,POPs通过竞争性抑制和变构等动力学和热力学过程改变酶、转录因子和GPCRs等蛋白质靶点的活性,调节细胞信号传导过程,导致炎症和癌症等多种病理变化^[65,66]^。因此研究污染物与蛋白质的相互作用是阐明污染物毒性机制的重要手段。POPs进入人体后,与之相互作用的第一类蛋白质是存在于胃中的消化酶,例如PFOA/全氟壬酸(PFNA)暴露导致胃蛋白酶构象发生变化^[67]^。POPs从胃吸收进入血液,与白蛋白和血红蛋白等血浆蛋白表现出高度的亲和力而阻碍了其生理功能,如杀螟丹与血红蛋白结合,改变血红蛋白构象,造成氧结合能力下降,导致高铁血红蛋白血症^[68]^。有些POPs与体内激素受体、GPCRs、转运蛋白和离子通道等具有高亲和力和特异性,如滴滴涕(DDT)的化学结构与激素相似,能与促卵泡激素受体(FSHR)相互作用,促进环磷酸腺苷(cAMP)的产生并破坏内分泌信号,可能对生殖和性发育产生不利影响^[69]^。有的POPs进入到细胞质中,进一步与核受体/转录因子(如芳香烃受体)相互作用,导致体内平衡破坏,甚至造成遗传毒性^[65]^。

### 2.4 污染物的联合毒性

化学品对健康的影响信息之前大多来自于流行病学和毒理学的研究,通过分析几种污染物与特定表型的关系,预测暴露-疾病关系中的机制。然而在日常生活中,大多数化学物质并非孤立存在,人体接触的是庞大的化合物种类,因此很难孤立地观察和明确一种化学物质的影响。很多情况下,暴露组学所关注的许多化学物质来自相同或相关的来源(如工业过程、消费品和饮食),这些化学物质在暴露和富集途径等方面常呈现相互关联的动态变化模式,这一特征与复杂生物系统中的群落结构类似。因此,通过对相关污染物进行分组可以降低混合污染物的复杂性,进而探索他们对生物体的影响^[70]^。但是,不同类型、不同来源污染物的联合效应则更复杂,污染物之间的相互作用可能会加剧他们在机体内的毒性^[71]^(见图3)。以塑料为例简要说明这个问题:塑料因其持久性、普遍性和潜在的毒性,已成为环境的主要威胁之一,预计到2050年,自然环境中将有约120亿吨塑料废物^[72]^。其中,微/纳米塑料(MPs/NPs)问题尤其引人关注,这类惰性有毒物质的粒径决定其容易通过消化道进入机体以及在机体内的分布^[73]^。MPs/NPs通过表面吸附和空隙填充装载各种污染物(如重金属、POPs、药物、细菌和病毒等)并将其输送、释放到新的地点(特别是生物体内)的特性被定义为“特洛伊木马效应”^[74,75]^。例如在肠道内环境条件下,会增加塑料中增塑剂双酚A(BPA)的释放,BPA进入细胞内可以与雌激素核受体相互作用,干扰内分泌系统的正常功能^[54,76]^。MPs/NPs的毒性效应不止来源于塑料本身和塑料添加剂,其吸附或负载的污染物对生物产生的综合毒理学效应涉及多个生命阶段,涵盖发育、行为、基因和代谢毒性、免疫反应和肠屏障功能障碍等诸多方面^[77⇓⇓⇓-81]^,已成为环境毒理学领域的研究热点之一。多数研究表明,与单个污染物相比,MPs-污染物混合物的毒性更强^[82]^。MPs/NPs联合全氟化合物(PFOA和PFOS)暴露,导致蚯蚓对PFOA和PFOS的吸收增加,并显著减少了蚯蚓的繁殖^[83]^。MPs暴露于人肝癌细胞株(HepG2)时,不会诱导细胞死亡,但当MPs与多氯联苯联合暴露时比多氯联苯单独暴露产生的毒性更大^[84]^。聚苯乙烯微塑料(PS-MPs)通过诱导线粒体去极化和抑制膜ATP结合盒(ABC)转运蛋白的活性,增加Caco-2细胞中砷的积累和毒性^[85,86]^。除MPs/NPs外,PM也可作为其他污染物的载体。PM是造成空气污染致病的元凶之一,接触大气PM与心血管和呼吸系统疾病相关的发病率和死亡率增加有关。据估计,全球每年有650万人死于空气污染^[87]^。近些年来,PM2.5对呼吸系统和心血管系统健康的影响引起了人们的广泛关注。PM2.5可沉积在整个呼吸道,特别是小气道和肺泡,导致肺功能降低,诱发哮喘和支气管炎,引发咳嗽和呼吸困难等症状,而且因其粒径小易被吸收,并且易携带重金属、多环芳烃和微生物等多种污染物质,对老人和小孩等敏感人群的作用尤为明显^[88,89]^。研究表明,单独暴露于甲醛或PM2.5对小鼠大脑几乎没有损伤,但对应剂量的甲醛与PM2.5联合暴露时,有显著的协同作用,加剧氧化应激和炎症,造成血脑屏障损伤、*β*-淀粉样蛋白1-42(A*β*_1-42_)和过度磷酸化的tau蛋白(p-tau)积累、胶质细胞激活等病理改变,导致认知功能下降^[90]^。

**图3:**
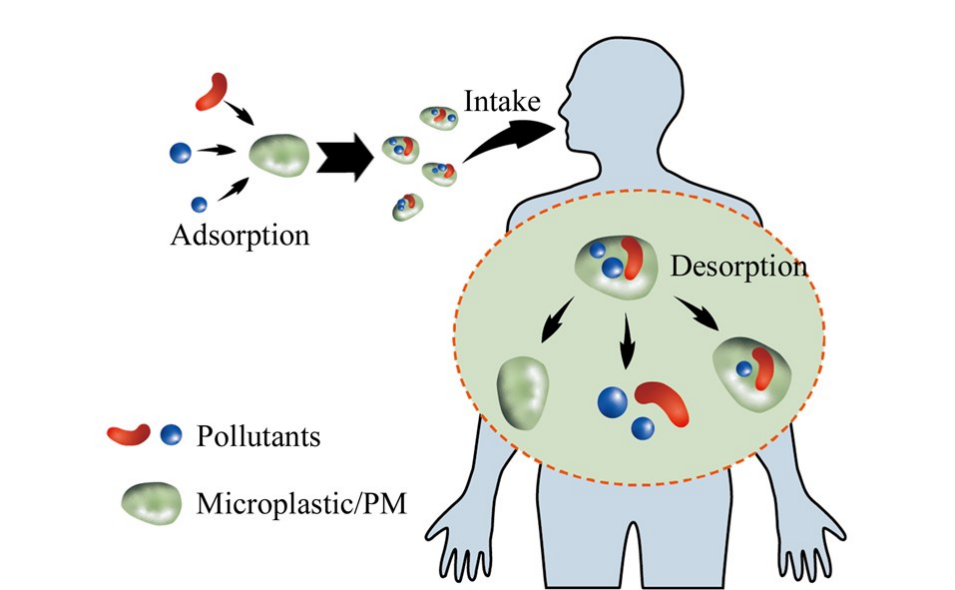
污染物的联合作用

## 3 总结

本文通过概括“环境-暴露组-健康”间的关系探讨了环境暴露对健康的影响。人们长期处于复杂的环境暴露中,需要我们综合考虑多种物质的共同作用,阐明重点污染物的健康效应、作用机制以及环境因素之间的相互影响,通过改变环境中的不利因素来减少疾病的发生。据估计,人类一生要经历超过100万次的暴露,因此认识生命不同阶段的暴露组及对健康的特定影响是一项巨大的挑战。首先,与基因组不同,暴露组在时间和空间上是高度可变的,这增加了描述他们对细胞、器官和生物体水平影响的难度;其次,表征未知分析物是了解暴露组的主要挑战。随着社会的发展,化学物质进入环境的速度、数量和种类都在不断扩大,绝大多数疾病的发生是由未知的暴露因素引起的;最后,找出暴露、暴露影响和其他因素(如遗传和疾病)之间的统计关联,需要整合大量内/外暴露检测数据。环境暴露的复杂性与我们目前的知识数据库之间存在巨大差距,丰富数据库任重而道远。
